# International Dental Federation: Meeting Held in Stockholm, Sweden, August, 1902

**Published:** 1903-07

**Authors:** 


### INTERNATIONAL DENTAL FEDERATION: MEETING HELD IN STOCKHOLM, SWEDEN, AUGUST, 1902.

(Concluded from page 468.)

### COMMISSION OF HYGIENE AND PUBLIC DENTAL SERVICES.

*Monday, August 18, 1902.*

THE opening session of the Commission of Hygiene and Public Dental Services was held in the Caroline Institute, Stockholm, Monday, August 18, 1902, with Professor Dr. Godon in the chair.

The following members were present: Drs. Queudot, Zsigmondy, Wachsmann, Lizka, Hesse, Davenport, Younger, Gordon White, Hayes, Viau, Martinier, Frick, B. Holly Smith, Godon, Guerini, Benson, Harding, Whittaker, Bain, Guldberg, Weber, Bensow, Smith-Housken, Barrett, Bödecker, Weiser, Förberg, Heidé, Frank, Jenkins, Cunningham, Röse, Sauvez, Aguilar, and Harlan.

The Commission then proceeded to the election of officers, the result of which follows: N. S. Jenkins, Dresden, president; Dr. C. Röse, Dresden, Dr. J. Frank, Vienna, Dr. E. Förberg, Stockholm, Dr. G. Cunningham, Cambridge, vice-presidents; Dr. R. Heidé, Paris, secretary.

Dr. J. Frank presented the report of the Commission of Hygiene, which was not read because of its considerable length.

Appended to this report was a communication (marked "A") of the Austrian Secretary of the Interior to the local authorities, instructing them to study the question of the relation of dentistry to public hygiene; one marked "B," comprising the sixteen questions that were addressed to the members of the Commission in different countries; one marked "C," being the report of Dr. Frank to the Austrian Secretary of the Interior, comprising besides the principal document several smaller ones; a report by Dr. E. C. Kirk, Philadelphia, on public dental hygiene in the United States, comprising the text of the law providing for dental services in the army, and an extract from the *Dental Cosmos* on the proposed law to provide for dental services in the navy; also several papers embodying the answers to the questions sent out by the special committee composed of Drs. Elof Förberg, George Cunningham, and Johan Frank. These answers refer to the condition of public dental hygiene in England, Denmark, Norway, Russia, and Switzerland.

The conclusions of the report are as follows:

1. Popular dental hygiene is as yet in a state of detrimental neglect in the majority of civilized States, though here and there are discovered evidences of an initial improvement.

2. The Executive Council will address through the national federations a memoir upon dental services to the different governments, inviting them to submit the propositions therein contained to competent examination.

Dr. Frank then read the circular which the committee proposed be sent to the governments of the various countries. The circular here follows:

The Executive Council of the International Dental Federation, at its session held in Stockholm, August 15 to 20, 1902, has discussed and approved the report of the Commission of Hygiene, and, following the suggestion therein embodied, has agreed to request the co-operation of the governments of the different States towards that branch of public hygiene which deals with the care of the mouth and teeth.

The Executive Council has learned with regret that this very important branch of general hygiene has been conspicuously neglected in practically all countries, and that the pupils of the primary and secondary schools, representing about twenty per cent. of the entire population, do not receive any dental care whatsoever.

The Executive Council of the International Dental Federation, comprising the delegates of all the dental associations of different States, being concerned in elevating the scientific status of dentistry and in introducing it into the realm of public hygiene, respectfully beg to draw your attention to the accompanying reports, which we earnestly request may be submitted to the consideration of the eminent personages having charge of your systems of education and public hygiene. These reports show that dental caries is a public evil which is daily increasing in magnitude, and that in certain countries from sixty to ninety per cent. of all the inhabitants of twenty years of age are sufferers from dental caries. Furthermore, these reports show that this widely propagated malady can become the cause of general organic disturbances, that it makes of the mouth a culture medium for the development of zymotic germs which if permitted to develop produce diseases acute and chronic (even tuberculosis), and that a certain number of persons thus affected are rendered physically incapable of entering the ranks of certain callings.

The Executive Council take the liberty of calling your attention to the urgent need of eradicating this evil for the sake of human welfare, and consider that the best means of combating it consists in the organization of public dental services.

As regards dental services in schools we will refer you to the appended reports marked "D," "E."

The gratuitous dental services for the poor could be arranged as follows:

(*a*) By the appointment of public dentists.

(*b*) By the organization of dental clinics in the public hospitals.

(*c*) By giving free dental attention to the poor in the dental schools under governmental control.

(*d*) By the appointment of dentists in all charitable institutions.

With reference to the establishment of dental services in the army and navy, we beg to call your attention to Chapter III. of the report.

As to the organization of dental services for employees of inferior grades, we offer the suggestion that a savings-fund be organized among the men for this particular purpose, and that in manufacturing institutions in which the teeth of the employees are exposed to noxious influences (phosphorus, mercury, acids) dentists should be attached to the establishment in order to carry out bimonthly examinations of the teeth and to give proper conservative treatment. These dentists should be under the supervision of the district physician and of the inspector of factories. It would be likewise advisable to organize dental services among railroad employees.

Finally, it is of urgent necessity that councillors having a dental education or a certain degree of familiarity with dental matters should be appointed to the different boards of health. To these members could be intrusted the study of dental problems as well as the promulgation of measures concerning the organization of dental services.

EXECUTIVE COUNCIL, *F. D. I.*

Mr. A. R. Bain, Melbourne, spoke on the ideal arrangement of placing dental hygiene under State control. Every dentist should work towards the attainment of this reform, as dentists alone can appreciate its vast importance. The secret of healthy teeth everybody will admit depends mainly on the care and attention given to them in early life. It becomes at once evident that in early life the teeth should be inspected at frequent intervals, and the best way of accomplishing it would be by placing dental inspection under the care of the State. We must not lose sight of the fact that public authorities must be careful of the hygiene of the people in view of maintaining the health of the nation, and therefore, with the same purpose in view of elevating and improving the race, the teeth should not be neglected. The question is not only of an individual character, but also of national import.

Mr. Bain suggested that to the communication proposed by Dr. Frank be appended a notice to be posted in all public schools, setting forth in simple terms the imperative necessity of brushing the teeth after meals, the reason for doing it, the advantages of this practice, the dates of eruption of the permanent teeth, the advantages to be derived from dental inspection, and the list of free dental dispensaries for the treatment of those who for financial reasons could not afford to have their teeth attended to in private offices.

Dr. C. Röse thought that while Dr. Frank's report was well framed it was not sufficiently clear, and made a motion to the effect that its adoption should be deferred to the next meeting of the Commission in Madrid.

Dr. Frank thereupon stated that it had taken him a year to collect the data necessary for the preparation of the report, and that it would mean a loss of time to defer its discussion to the Madrid meeting.

Dr. Godon suggested that the conclusions alone be discussed at that session.

Upon motion by Dr. Sauvez, the Executive Council decided to undertake the study and final adoption of the report and the forwarding of it to the different governments.

The Commission then extended a vote of thanks to the members responsible for the preparation of the report, Drs. Frank, Cunningham, and Förberg.

Dr. Jenkins thought that the president of the Commission should be familiar with the French language, and as his own knowledge of that language is very imperfect respectfully submitted his resignation in favor of some one better suited for the office.

The members of the Commission, however, insisted upon having him as their chairman, and rejected his resignation by acclamation.

The meeting then adjourned.

*August 19, 1902.*

The meeting was called to order at the Grand Hotel by the president, Dr. N. S. Jenkins, Dresden.

Dr. Röse made a motion to the effect that a committee be appointed to correct the report as presented by Dr. Frank.

Dr. Heidé stated that, inasmuch as the report had been in the hands of the members for several days, they ought to be in a position to present their remarks at once.

Drs. Frank and Röse called attention to a few inaccuracies in the report.

Dr. Jenkins moved that the original report of the Commission be revised, translated, and addressed to each member of the Council, to be approved and signed before October 31, 1902.

The memorial presented by Dr. Frank on the previous day was then adopted.

Dr. Röse made a proposition with reference to the organization of conferences on public dental hygiene, saying that a good method of developing this important branch of general hygiene would consist in illustrating these lectures by means of appropriate slides, as carried out at the present time in Dresden.

Dr. Förberg said that, at Stockholm, illustrated lectures on dental hygiene have already been given by himself.

Dr. Heidé stated that also in France illustrated lectures have been given upon this topic.

After a few remarks by Dr. George Cunningham, the Commission adjourned to meet in Madrid in April, 1903.

